# Verification of Mechanical Properties Identification Based on Impulse Excitation Technique and Mobile Device Measurements

**DOI:** 10.3390/s23125639

**Published:** 2023-06-16

**Authors:** Lukasz Scislo

**Affiliations:** Faculty of Electrical and Computer Engineering, Cracow University of Technology, Warszawska 24, 31-155 Cracow, Poland; lukasz.scislo@pk.edu.pl

**Keywords:** vibration measurements, smartphone, mobile device, IET, impact excitation technique, material testing, mechanical properties, frequency response, Industry 4.0

## Abstract

The Impulse Excitation Technique (IET) is one of the most useful testing methods for evaluating or calculating some material properties. This can be useful to evaluate and confirm that the material ordered is what was delivered. In the case of unknown materials, where their properties are required by simulation software, this is also a quick way to obtain mechanical properties and thus improve the simulation quality. The main drawback of the method is the requirement for a specialized sensor and acquisition system and a well-trained engineer to prepare the setup and analyze the results. The article evaluates the possibility of using a low-cost solution in the form of a mobile device microphone as a way to obtain data, which after the Fast Fourier Transform (FFT), allows to obtain frequency response graphs and use the IET method procedure to calculate the mechanical properties of the samples. The data obtained by the mobile device are compared with the data obtained by professional sensors and data acquisition systems. The results confirm that for typical homogenous materials, the mobile phone is a cheap and reliable alternative for fast, on-the-go material quality inspections and can be introduced even in small companies and on construction sites. Additionally, this kind of approach does not require specific knowledge of sensing technology, signal treatment, or data analysis and can be performed by any assigned employee, who can receive the quality check information immediately on-site. Additionally, the presented procedure allows data collection and transfer to the cloud for future references and additional information extraction. This element is fundamental for introducing sensing technologies under the Industry 4.0 concept.

## 1. Introduction

Modern manufacturing, which is more focused on gathering information under Industry 4.0 concept, relies on fast and reliable measuring techniques. In choosing and ordering materials suitable for a given task, such as the construction of machine parts, manufacturing construction elements for civil engineering structures, or machining the replacements of given elements, material properties are usually the most important factor. This is because material properties are important for a variety of purposes. In the case of design and engineering, material properties help engineers and designers select the right materials for a particular application based on factors such as strength, stiffness, toughness, durability, and other mechanical, thermal, and chemical properties. In manufacturing and production, material properties influence how materials are processed, molded, machined, or formed during manufacturing. A complete understanding of the material properties can help ensure that the manufacturing process is efficient and effective and produces high-quality products. This is especially important, among others, for sheet forming [1], powder forming [2], and injection molding [3]. Furthermore, in quality control: material properties are used to measure and verify the quality of materials used in products. This helps to ensure that the products meet the required standards and are fit for their intended purpose. Moreover, for ensuring safety and reliability, material properties are crucial parameters to evaluate structures and systems [4,5]. Knowing the properties of materials used in critical applications such as aerospace, automotive, or medical devices can help prevent failure or accidents. Finally, those parameters are key issues in research and development, especially for researchers developing new materials or improving existing ones. Understanding the properties of materials helps researchers to identify new applications, improve performance, and develop new technologies. This is especially important nowadays in the case of nanostructured materials and nanocomposites [6], biomaterials [7], or when many materials are being developed using, e.g., additive manufacturing techniques [8,9,10] or continuous extrusion forming [11].

There are many material properties that can be considered when choosing the material for a specific task. However, elastic properties are one of the most important ones for fast identification of the given material and use as main input information for simulation tools. In general, the elastic properties of a material are the properties that describe how a material responds to an applied stress or strain within the elastic limit. The most commonly used elastic properties include:

- Young’s modulus: measures the material’s stiffness and resistance to deformation under tension or compression.
- Poisson’s ratio: describes the lateral deformation of a material in response to an applied axial load.
- Elastic limit: the maximum stress that a material can withstand before it becomes permanently deformed.
- Modulus of resilience: the energy required to deform a material to its elastic limit.
- Modulus of toughness: the energy required to break a material.

Moreover, elastic properties are often given together with the Poisson ratio, which specifically describes the lateral deformation of a material in response to an applied axial load. Poisson’s ratio is defined as the ratio of the lateral strain to the axial strain or the change in one dimension of a material relative to the change in the other dimension when it is subjected to an axial load. It must be pointed out that elastic properties and Poisson’s ratio are related but distinct mechanical properties of materials. Thus, elastic properties describe a material’s general response to stress and strain, while Poisson’s ratio is a specific property that quantifies the lateral deformation of a material under axial loading.

These properties are important in the design of structures and mechanical components to ensure that the material can withstand the loads and strains it will be subjected to during use. This is also a crucial issue in material selection electromechanical systems, especialaly in robotics [12,13], biomedical applications [14], power electronics [15,16], and optoelectronics [17].

**Figure 1:**
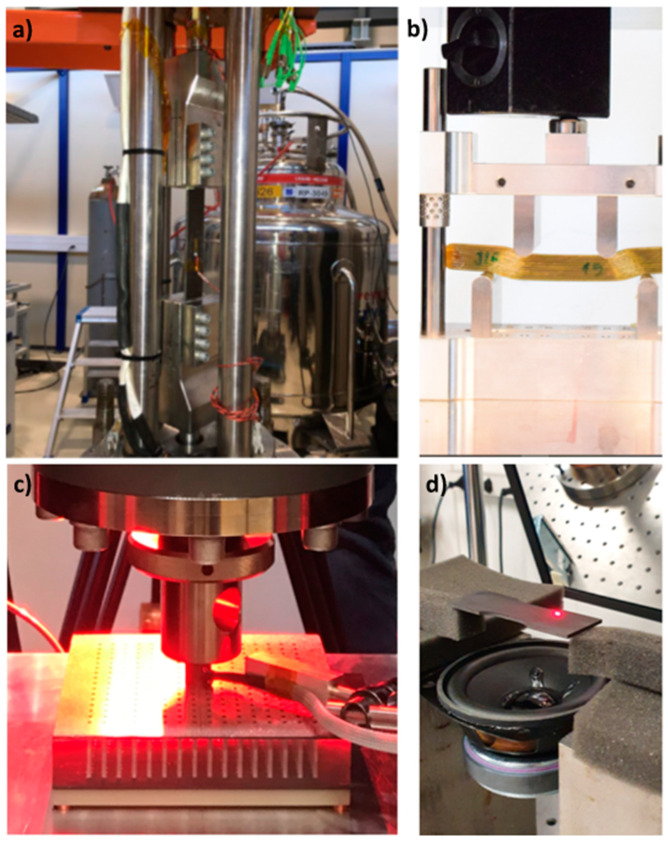
Typical material tests performed in the laboratory conditions, examples from Mechanical Measurement Laboratory at CERN. (**a**) Tensile test at cryogenic temperatures; (**b**) flexure test on composite material; (**c**) rigidity test with the universal testing machine; (**d**) dynamic testing with 3D Laser Doppler Vibrometry.

There are several methods for testing the elastic properties of materials, including tensile testing, compression testing, bend and rigidity testing, hardness testing, and Dynamic Mechanical Analysis (DMA). The tensile testing is a common method for measuring the elastic properties of materials. In a tensile test, a sample of the material is subjected to a tensile load until it fractures or reaches its elastic limit. The deformation and stress-strain behavior of the material can then be measured and used to determine properties such as Young’s modulus, ultimate tensile strength, and yield strength (Figure 1a). Compression testing is similar to tensile testing, but the sample is subjected to compressive loads instead of tensile loads. Compression testing is used to determine the compressive strength and stiffness of materials. During bend testing, a sample of the material is bent until it fractures or reaches its elastic limit. The deformation and stress-strain behavior of the material can then be measured and used to determine properties such as flexural strength and modulus of elasticity (Figure 1b). Rigidity testing, on the other hand, is a type of test that is used to determine the stiffness or rigidity of a material. In a rigidity test, a sample of the material is subjected to a bending force, and the resulting deflection is measured. The rigidity of the material is determined by calculating the modulus of elasticity or Young’s modulus, which is a measure of the material’s stiffness (Figure 1c). Moreover, dynamic DMA is a method for measuring the mechanical properties of materials as a function of frequency and temperature. DMA is a technique used to evaluate the viscoelastic properties of materials, which includes both the elastic and viscous behavior of the material under stress. Furthermore, there are numerous methods of indirectly measuring the elastic properties of materials. Hardness testing is a measure of the material’s resistance to permanent deformation and is often used as an indicator of the material’s elastic properties. Another possible solution is to use sophisticated equipment used for other tasks but allows to obtain the data that can be used to calculate elastic properties. One example is the Laser Doppler Vibrometer which usually is used for modal or operational modal analysis [18] or quality control [19,20], but with a connection to, e.g., the IET method can also be used to calculate elastic properties (Figure 1d).

The main problem with elastic properties testing is the need for an expensive experimental setup or specialized machines as well as qualified personnel for sample preparation, mounting, setting up the equipment parameters, and data evaluation. This requires the typical tests to be mostly performed in laboratory conditions.

One of the other possibilities that are often used is the simple acoustic speed-elasticity relationship or natural frequencies–elastic properties relationship. In the first scenario, the ultrasonic methods are being used [21], and in the second one, the so-called Impulse Excitation Method (IET) [22].

IET is one of the less expensive techniques that can be considered for a quick and accurate way to obtain such properties as Young’s modulus, shear modulus, and Poisson’s ratio. In the case of this method, after the application of force to a well-described sample (mass and dimensions), the frequency response function is obtained and used for specific parameter calculations. This method can be used for fast quality assurance purposes to check if the material ordered is indeed the material received. Moreover, the test can be used as an alternative to tensile tests for the identification of damaged samples, e.g., composite laminates [23,24]. Additionally, typically to obtain the elastic properties of a sample while heating or cooling, a thermomechanical analysis (TMA) test is used. However, it is also possible to use the internal friction calculation (obtained by impulse excitation techniques) and evaluate the heat treatment quality of metals or ceramics [25] or during the gel casting process, which is used to obtain ceramic elements with a designed shape [26]. Furthermore, depending on the data needed, the IET measurements also allow the evaluation of internal friction and damping properties of a given material [27]. Moreover, the usefulness of the method is not limited to one industry. It can be introduced to evaluate medical products and medicine (tablets) [28,29].

It must be noted that this method can be applied as full none contact where both the measurement (using a microphone) and the excitation (e.g., sound or air pressure) are performed without direct influence on the sample surface. This is especially beneficial in evaluating light and ultra-light materials where touching or adding mass to the structure will influence the results. Thus, the acquisition of response function using the IET method is well established in the aerospace industry, where the need to test fragile, lightweight structures is especially useful [30,31]. In the case of such measurement, sophisticated equipment, such as a 3D Laser Doppler Vibrometer, is used to perform IET measurements (Figure 1d) [18,32].

The IET method, due to its flexibility in measurement techniques, is also a good fit for Industry 4.0 applications where technologies such as the Internet of Things (IoT), cloud computing, and big data analytics are being integrated into manufacturing processes. Using vibration measurement sensors as IoT devices is an emerging field. This approach is becoming widely used in the monitoring of construction-induced vibration monitoring [33], health monitoring of civil engineering structures [34], predictive maintenance during the manufacturing process [35], and quality control [19,20]. Regardless of the application, the vibration data is sent to cloud storage for analysis and future information extraction. This allows us to use this data for additional purposes and find ways to automate processes and reduce costs.

The main objectives of the paper are as follows:

(1) Finding a low-cost solution to perform quality assurance tests of materials without needing sophisticated measurement equipment, such as universal testing machines, laser vibrometers, professional sensors (e.g., accelerometers, microphones), and acquisition systems together with the licensed software. The typical equipment used also requires a well-trained engineer to prepare the setup, adjust signal acquisition parameters, and analyze the data.
(2) Present the possibility of using a mobile device with its sensor (microphone) as a reliable tool for obtaining materials’ elastic properties.
(3) Present a typical application and advantages of using such a setup for IET method analysis on typical materials used for the construction of technical objects.
(4) Show that the IET method, using a smartphone as a sensing solution and a way to analyze the data quickly, allows for its direct implementation even in small companies with no sensing equipment resources.

Thus, the paper presents the results of using the IET procedure on several typical materials used in the construction of machines, structures, or daily life equipment. Additionally, the Industry 4.0 concept can transfer the data immediately after the test to the cloud for additional processing or simple historical data analyses.

## 2. Materials and Methods

The purpose of vibration measurements, depending on the needs, is to obtain the amplitude of the given parameter (e.g., acceleration) over time or Frequency Response Function (FRF) in case of evaluation of data in the frequency domain [36].

**Figure 2:**
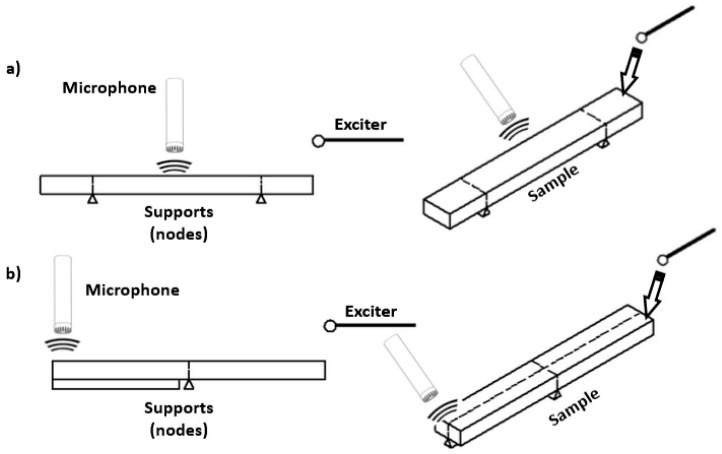
IET measurement principle: (**a**) obtaining the first flexural mode; (**b**) Obtaining the first torsional mode of the rectangular cross-section sample.

Among many possible techniques and solutions, the IET technique seems especially profitable in the case of fast quality control of materials. This test method covers the determination of the dynamic elastic properties of elastic materials at ambient temperatures. Specimens of these materials possess specific mechanical resonant frequencies that are determined by the elastic modulus, mass, and geometry of the test specimen. The dynamic elastic properties of a material can therefore be computed if the geometry, mass, and mechanical resonant frequencies of a suitable (rectangular or cylindrical geometry) test specimen of that material can be measured. Dynamic Young’s modulus is determined using the resonant frequency in either the flexural or longitudinal mode of vibration. The dynamic Shear modulus, or modulus of rigidity, is found using torsional resonant vibrations. Dynamic Young’s modulus and dynamic shear modulus are used to compute the Poisson’s ratio. Depending on the requirement to find a specific flexural or torsional mode, a specific arrangement of supports, sensors, and exciter is required (Figure 2).

The specific material properties are calculated using the following equations. The dynamic Young’s modulus is determined using the 1st flexural resonant frequency and Equation (1).

(1) $$E=0.9465(\frac{mf_{f}^{2}}{b})(\frac{L^{3}}{t^{3}})T_{1}$$

where

E—Young’s modulus, Pa
m—a mass of the sample (a bar), g
b—width of the sample (a bar), mm
L—length of the sample (a bar), mm
t—thickness of the sample (a bar), mm
$f_{f}$—fundamental resonant frequency of sample (a bar) in flexure, Hz
$T_{1}$—correction factor for fundamental flexural mode to account for finite thickness of the sample (a bar), Poisson’s ratio, and so forth

If $\frac{L}{t}\geq 20$, $T_{1}$ can be simplified into the following

(2) $$T_{1}=[1.000+6.585{\left(\frac{t}{L}\right)}^{2}]$$

The dynamic shear modulus is determined using the first torsional resonant frequency using Equation (3).

(3) $$G=\frac{4Lmf_{t}^{2}}{bt}R$$

where

G—dynamic shear modulus, Pa,
$f_{t}$—fundamental resonant frequency of sample (a bar) in torsion Hz.

(4) $$R=\left[\frac{1+{\left(\frac{b}{t}\right)}^{2}}{4-2.521\frac{t}{b}(1-\frac{1.991}{e^{\pi \frac{b}{t}}+1})}\right]\left[1+\frac{0.00851b^{2}}{L^{2}}\right]-0.060{\left(\frac{b}{L}\right)}^{\frac{3}{2}}{\left(\frac{b}{t}-1\right)}^{2}$$

Dynamic Young’s modulus and dynamic shear modulus are used to compute the Poisson’s ratio using Equation (5).

(5) $$\mu =\frac{E}{2G}-1$$

**Figure 3:**
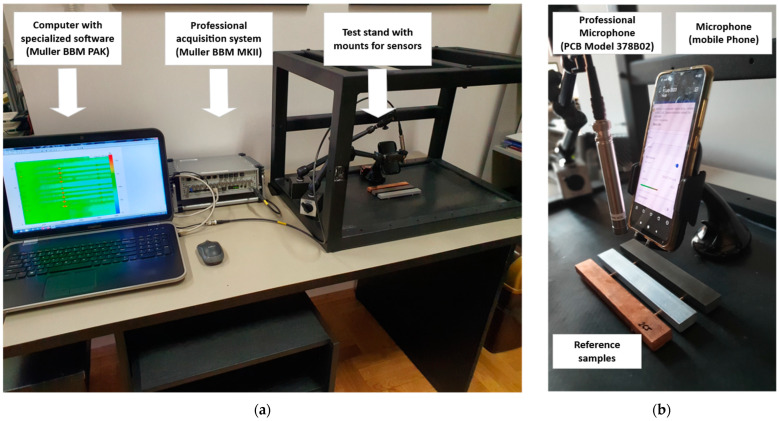
Test stand overview: (**a**) the view of the complete test stand; (**b**) the view of the sensors and test samples.

In the case of typical usage of the IET method, a laboratory setup is required. It consists of a professional ICP sensor (e.g., microphone PCB Model 378B02) and the acquisition system (e.g., Muller BBM MKII or MicroQ). Additionally, specialized software is usually required. However, for most cases, modern mobile phone sensors may be an alternative solution allowing one to obtain and display the FRF function without a sophisticated setup. The comparison of the professional sensor and mobile phone sensor can be seen in Table 1. For testing purposes, a mobile phone Samsung Galaxy S10 was used and compared with a professional laboratory setup (Figure 3).

The Samsung Galaxy S10 features three microphones, two at the bottom and one at the top. These microphones provide audio input for features such as phone calls, voice recordings, and voice commands. The specific specifications of the microphones in the Galaxy S10 are not publicly disclosed by Samsung. The sensitivity of a microphone is usually measured in decibels of sound pressure level (dB SPL) per millivolt (mV) of output. The sensitivity of a phone microphone can range from about −40 to −60 dB SPL, with a typical value of around −45 dB SPL.

Finally, three test samples were chosen for the test. The materials chosen are of well know properties and have a wide range of usage in engineering structures and machines. It was decided to test samples of aluminum (Young’s modulus usually E ≈ 65–75 GPa depending on the alloy), steel (Young’s modulus E ≈ 190–200 GPa depending on the specific type of steel), and copper (Young’s modulus E ≈ 100–140 GPa depending of the alloy).

## 3. Results

During the experimental test, the different materials were tested with multiple force impulses applied to each of the samples.

**Figure 4:**
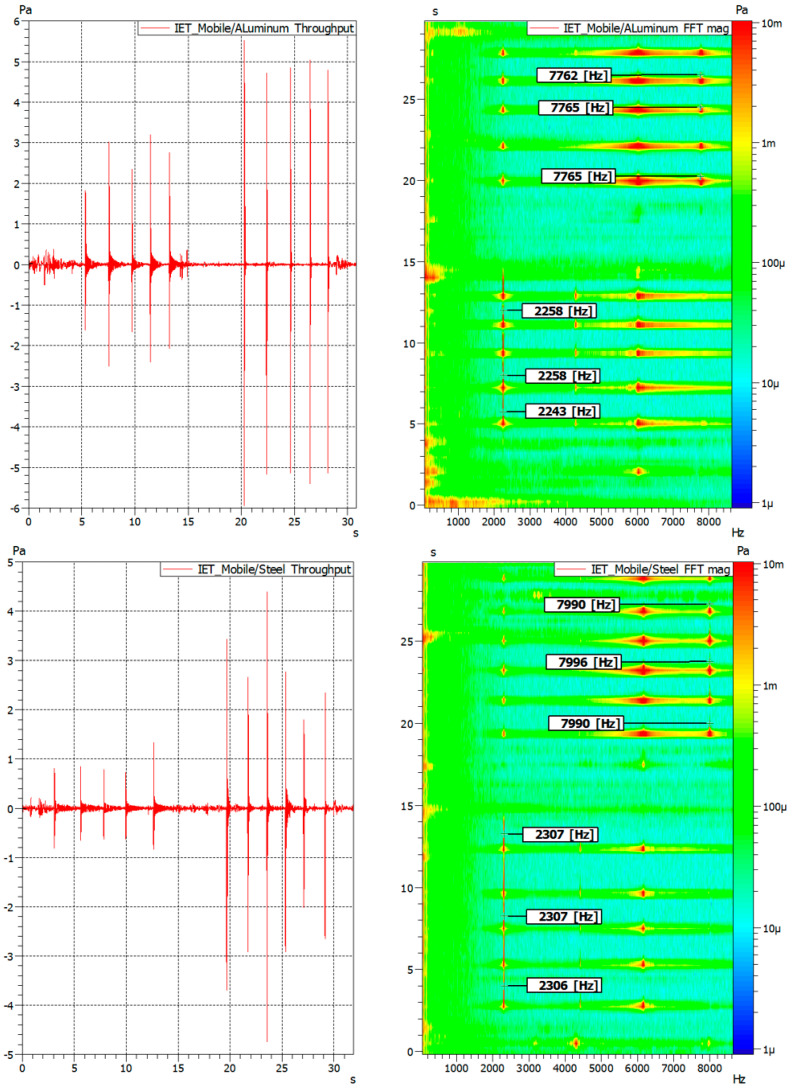

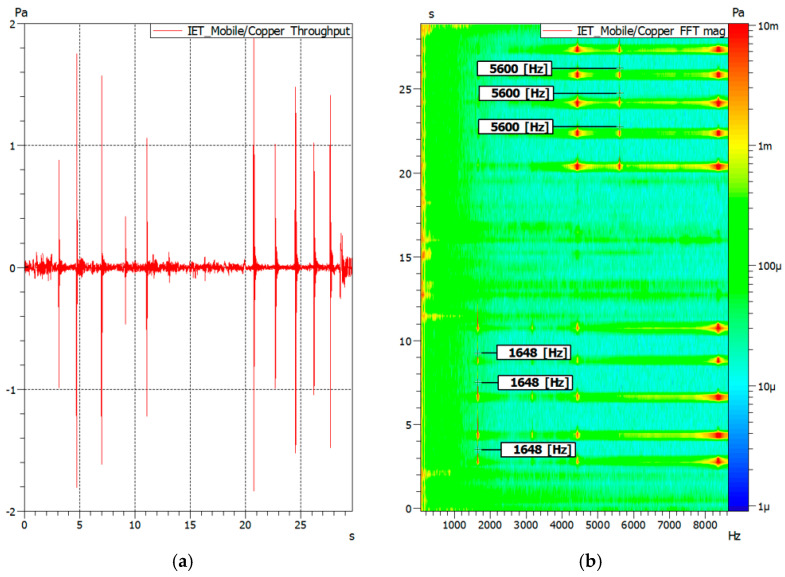
Results of frequency response analysis on the aluminum, steel, and copper samples (**a**) time data; (**b**) frequency data (3D FFT magnitude graph).

Figure 4 presents the FFT magnitude graph in the form of a so-called waterfall or rain chart. The FFT rain graph is a 3-dimensional representation of the frequency distribution of the amplitude over time. To make the identified resonant frequencies even more visible, the color contrast is used to present in which frequency the given vibration form is excited. Additionally, in the first 15 s, the sample was arranged in the setup to excite the flexural modes (Figure 2a). Then, the supports and microphone placement were rearranged to excite the torsional modes (Figure 2b). This is clearly visible in Figure 4, where detected natural frequencies for the first flexural and the first torsional modes were marked together with the value associated with maximal amplitude.

The values presented in Figure 4 were used to calculate the elastic properties of all three samples (Table 2). It must be mentioned that the sample size was constant due to control during sample manufacturing and specialized surface preparation. To minimize the effect of the error, each sample was excited multiple times, and the average for all the tests was calculated. The eventual difference in first flexural or torsional frequencies was minimal and in the ranges of a few Hz.

The values obtained and calculated using the IET procedure corresponded to table values for aluminum, steel, and copper. Thus, the measurement using professional equipment was verified.

The same procedure was used to test samples with the mobile device microphone sensor, according to the test setup presented in Figure 3. The results of the measurements and calculations are presented in Table 3.

**Figure 5:**
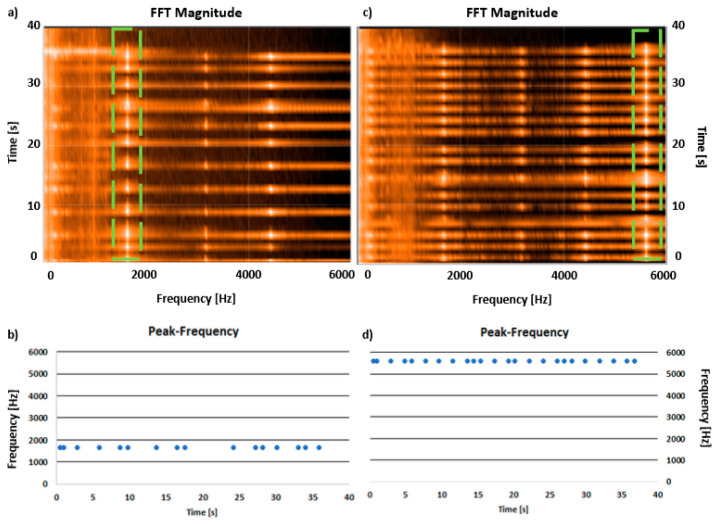
Results of frequency response analysis on the copper sample by a mobile phone. (**a**) FFT magnitude for support setup to obtain flexural modes; (**b**) dominant frequency registered for each force impulse (blue points) for support setup to obtain flexural modes; (**c**) FFT magnitude for support setup to obtain torsional modes; (**d**) peak-frequency registered for each force impulse (blue points) for support setup to obtain torsional modes.

In the case of the mobile device measurements, the specific resonant frequencies can be identified immediately using a graphical interface (Figure 5a,c) or after sending to the cloud storage and identifying the dominant frequency from the raw data (blue points in Figure 5b,d).

## 4. Discussion

Both the graphical interface and the exported data allow identifying of the required frequencies. Similar to measurements performed by the professional system, it is again easy to identify the first flexural (Figure 5a—green line) and after changing the setup first torsional modes (Figure 5c) from the graphical interface after the measurement performed with the mobile device. Additionally, looking at the peak-frequency data for each of the force impulses (Figure 5b,d) during mobile device measurements, it is possible to conclude that the measurement stability is high. There are no false frequencies detected, and the values of each frequency do not differ more than a few Hz.

Taking into account the measurement values of the frequency response and considering the inherent uncertainties associated with the measurement technique and instrument calibration, the percentage difference in the case of the first flexural and the first torsional resonant frequencies, as well as for calculation of the dynamic Young’s modulus and the dynamic shear modulus, the acquired percentage difference was, for most cases, below 1%. Table 4 presents the averaged values from the measurements performed by both evaluated systems, together with the calculated percentage differences.

As presented, according to the method, the test specimens of suitable geometry are excited mechanically with a singular elastic strike using an impulse tool to measure the fundamental resonant frequency. In order to convert the mechanical vibrations of the specimen into electrical signals, a transducer (such as a contact accelerometer or non-contacting microphone) is used. However, the additional possibility of using a fully non-contact method is also possible with the use of, e.g., a 2D or 3D Laser Doppler Vibrometer to obtain a frequency response function at a given point. Depending on the chosen method following elements are crucial:

- Using a contact setup with a transducer (accelerometer) glued to the surface and excitation from impulse, e.g., modal hammer or shaker, is allowed only for large, heavy structures due to added mass from the transducer. For smaller samples and lightweight materials, using even a small accelerometer of a few grams will introduce an error in the measurements. This error will be even more significant while calculating Poisson’s ratio due to its value. This method requires investments in the equipment (sensors, exciters), specialized software, and a well-trained engineer to prepare the setup, choose correct acquisition parameters, and analyze the data.
- Using the semi-contact method with the use of a contactless transducer (microphone and contact impulse, as presented in the control example in this study (Figure 3a), limits the problem of changing the structure dynamics due to changes in the object mass. However, the problem of the cost of the measuring equipment, the time needed for the test preparation, and personal costs is still a significant issue.
- Using a fully non-contact method with the use of, e.g., a 3D Laser Doppler Vibrometer as sensing equipment and the loudspeaker as an element introducing energy to the system, allows for reliable measurements with high accuracy (Figure 1d), as the author presented in previous works [18,19]. Additionally, due to the possibility of visualizing the forms straight after the test and thus identification of both the first flexural and first torsional frequencies, it is possible to perform the test without the need of changing support conditions or in the free-free conditions, e.g., using elastic strings. This makes this technique especially suitable for the measurement of light and ultralight materials or samples of fragile materials. Although the method is the most advanced, it has significant drawbacks in case of potential use. Due to the system’s cost, it is rarely used for similar tests authorized by the aerospace or automotive industries or highly specialized laboratories, such as the Mechanical Measurement Laboratory at CERN. Due to the cost and optical component, it is problematic to use for simple field tests, such as IET measurements of materials shipments. It also requires highly trained engineers for preparing the measurement, as well as the need for creating a new setup for every sample.
- Using the proposed method with the use of a smartphone’s microphone as a transducer and a small hammer or even a hand-crafted exciter, as presented above (Figure 3b), allows for reliable results comparable with professional equipment. It can be performed without laboratory conditions and sophisticated equipment. The results are obtained immediately after the test, and the system does not require specialized knowledge; thus, it is ideal for on-site material quality checks. Opposite to using sophisticated optical equipment, such as a 3D Laser Doppler Vibrometer, it is possible to measure multiple samples one after another without downtime needed for individual preparation of the measurement setup. Thus, multiple samples can be measured in a matter of minutes. Moreover, the cost of the whole measurement system contains the cost of the smartphone itself. Finally, due to its universality, the user is not limited by anything and can stream data to the cloud, incorporate measurements with companies software, e.g., Product Lifecycle Management (PLM), Product Data Management (PDM), Enterprise Resource Planning (ERP), Manufacturing Execution System (MES), and others, or connect the data stream with simulation software if the aim is to obtain reliable data, e.g., Finite Element Method (FEM) simulations.

## 5. Conclusions

The results of this study unequivocally support the initial hypothesis put forth, indicating a clear validation of the proposed hypothesis that a mobile phone can be successfully used as a sophisticated measurement system for dynamic measurements. Especially, in the case of the IET method, usually used to check the quality of supplied materials, it gives values of mechanical properties for computer simulations or identifies the material based on the dynamic Young’s and shear modulus. The use of the mobile device is advantageous. It allows the fast measurement and data analysis without sophisticated equipment (sensors, data acquisition system, specialized software), cables, power supply etc. Moreover, each of those elements may be the source of additional measurement errors. Finally, the data from the mobile device can be visualized immediately using a graphical interface and be stored locally in case of a lack of an internet connection, but it can also be transferred to the cloud for in-depth analysis and storage. Thus, the mobile device becomes an IoT measurement system with a cloud component.

The data collected and analyzed in this paper provide robust evidence that aligns with the anticipated outcomes, thus affirming the validity of our original conjecture that the mobile device can successfully take over expensive professional equipment in the case of such measurements.

## Figures and Tables

**Table 1:** Test sensors specification.

Property	Microphone PCB Model 378B02	Microphone Mobile Phone *
Sensitivity [±1.5 dB]	−26	−40
Sensitivity [mV/Pa]	50	10
Frequency Range [Hz]	3.75 to 20,000	20–20,000

* Approximate data based on typical sensor properties. Details depend on the specific phone and are not publicly disclosed.

**Table 2:** IET measurements and calculations results using professional test equipment.

Sample	Flexural Resonant Frequency f_f_ [kHz]	Torsional Resonant Frequency f_t_ [kHz]	Mass	L [mm]	b [mm]	t [mm]	E [GPa]	G [GPa]	μ [-]
Aluminum									
Measurement 1	2.258	7.762	81.5	150	20	10	66.372	26.779	0.239
Measurement 2	2.258	7.765	81.5	150	20	10	66.372	26.800	0.238
Measurement 3	2.243	7.765	81.5	150	20	10	65.493	26.800	0.222
**Average**	**2.253**	**7.764**	**81.5**	**150**	**20**	**10**	**66.079**	**26.793**	**0.233**
Steel									
Measurement 1	2.307	7.990	236	150	20	10	200.625	82.166	0.221
Measurement 2	2.307	7.996	236	150	20	10	200.625	82.289	0.219
Measurement 3	2.306	7.990	236	150	20	10	200.451	82.166	0.220
**Average**	**2.307**	**7.992**	**236**	**150**	**20**	**10**	**200.567**	**82.207**	**0.220**
Copper									
Measurement 1	1.648	5.600	265	150	20	10	114.958	45.322	0.268
Measurement 2	1.648	5.600	265	150	20	10	114.958	45.322	0.268
Measurement 3	1.648	5.600	265	150	20	10	114.958	45.322	0.268
**Average**	**1.648**	**5.600**	**265**	**150**	**20**	**10**	**114.958**	**45.322**	**0.268**

**Table 3:** IET measurements and results obtained using a mobile phone microphone.

Sample	Flexural Resonant Frequency f_f_ [kHz]	Torsional Resonant Frequency f_t_ [kHz]	E [GPa]	G [GPa]	μ [-]
Aluminum					
Measurement 1	2.247	7.783	65.727	26.924	0.221
Measurement 2	2.244	7.781	65.551	26.910	0.218
Measurement 3	2.243	7.781	65.493	26.910	0.217
**Average**	**2.245**	**7.782**	**65.590**	**26.915**	**0.218**
Steel					
Measurement 1	2.291	7.995	197.851	82.269	0.202
Measurement 2	2.294	7.994	198.370	82.248	0.206
Measurement 3	2.294	7.995	198.370	82.269	0.206
**Average**	**2.293**	**7.995**	**198.197**	**82.262**	**0.205**
Copper					
Measurement 1	1.646	5.595	114.679	45.241	0.267
Measurement 2	1.645	5.596	114.539	45.257	0.265
Measurement 3	1.646	5.596	114.679	45.257	0.267
**Average**	**1.646**	**5.596**	**114.632**	**45.252**	**0.267**

**Table 4:** Percentage difference calculation for the averaged measurement values acquired in the case of measurements with a professional system and a mobile phone.

Sample	Muller BBM	Mobile Phone	Difference
Aluminum			
Flexural Resonant Frequency $f_{f}$ [kHz]	2.253	2.245	**0.36%**
Torsional Resonant Frequency $f_{t}$ [kHz]	7.764	7.782	**0.23%**
E [GPa]	66.079	65.590	**0.74** **%**
G [GPa]	26.793	26.915	**0.45** **%**
Steel			
Flexural Resonant Frequency $f_{f}$ [kHz]	2.307	2.293	**0.61** **%**
Torsional Resonant Frequency $f_{t}$ [kHz]	7.992	7.995	**0.04** **%**
E [GPa]	200.567	198.197	**1.19** **%**
G [GPa]	82.207	82.262	**0.07** **%**
Copper			
Flexural Resonant Frequency $f_{f}$ [kHz]	1.648	1.646	**0.12** **%**
Torsional Resonant Frequency $f_{t}$ [kHz]	5.600	5.596	**0.07** **%**
E [GPa]	114.958	114.632	**0.28** **%**
G [GPa]	45.322	45.252	**0.15** **%**

## Data Availability

Not applicable.
